# An Approach to Localizing Corneal Pain Representation in Human Primary Somatosensory Cortex

**DOI:** 10.1371/journal.pone.0044643

**Published:** 2012-09-04

**Authors:** Eric A. Moulton, Lino Becerra, Perry Rosenthal, David Borsook

**Affiliations:** 1 P.A.I.N. Group, Center for Pain and the Brain, Children's Hospital Boston, Massachusetts General Hospital, McLean Hospital, Harvard Medical School, Boston, Massachusetts, United States of America; 2 Boston Foundation for Sight, Needham, Massachusetts, United States of America; Johns Hopkins University, United States of America

## Abstract

The cornea has been a focus of animal electrophysiological research for decades, but little is known regarding its cortical representation in the human brain. This study attempts to localize the somatotopic representation of the cornea to painful stimuli in human primary somatosensory cortex using functional magnetic resonance imaging (fMRI). In this case study, a subject was imaged at 3T while bright light was presented in a block-design, which either produced pain and blinking (during photophobia) or blinking alone (after recovery from photophobia). Pain and blinking produced precisely localized activations in primary somatosensory cortex and primary motor cortex. These results indicate that noxious stimulation of the cornea can produce somatotopic activation in primary somatosensory cortex. This finding opens future avenues of research to evaluate the relationship between corneal pain and central brain mechanisms relating to the development of chronic pain conditions, such as dry eye-like symptoms.

## Introduction

The cornea is densely innervated by A-delta and C-fiber primary afferents [1], [2], [3]. These may be directly imaged in humans using *in vivo* confocal microscopy to evaluate their structure in normal and pathological conditions [4]. Despite the high density of innervation, no data are available regarding the representation of corneal sensation in the primary somatosensory cortex (S1). Localization of the cortical representation of this structure would open new avenues of research to evaluate peripheral-central interactions of sensory encoding in human volunteers.

Neuroimaging investigations to evaluate how corneal pain may affect brain systems may contribute to an improved understanding of the development of neuropathic pain from peripheral damage to the eye. Corneal pain is a significant clinical problem, with more than 20 million patients suffering from chronic dry eye-like corneal symptoms in the U.S. alone (Market Scope 2011 – Comprehensive Report on the Global Dry Eye Market). The human cornea is especially favorable to study neuropathic pain because of its discrete, high density of free nerve endings, transparency, accessibility to topical treatments, and its role as a major part of the trigeminal system. Demonstration of the feasibility of functional neuroimaging to localize the cornea in S1 is the first step in developing an eye-to-brain model to study chronic pain. Here, we report the probable localization of the cortical representation of the cornea in the somatosensory homunculus by evaluating cortical activation evoked by (1) acute pain localized to the cornea, and (2) involuntary blinking of the eyelids as a parallel motor control.

## Methods

In order to evaluate the potential location of the cornea in S1, we evaluated data obtained fMRI from a patient with and without corneal pain that resulted from hard-contact lens abrasion to the left eye. The subject was a right-handed 54-year old male. This “n of 1 study” met the approval of the McLean Hospital Institutional Review Board, and the subject provided written informed consent as part of a developmental protocol.

The subject dataset and analysis methods in this paper were previously described in a case report on reversible sensitization of a trigeminal nociceptive pathway in photophobia [5]. Briefly, the subject underwent two fMRI scans: one while afflicted with photophobia (pain induced by light), and another after recovery 9 days later. For each scan session, the subject was presented a white fixation cross on a black background at rest, interspersed with nine blocks of a white background (6 sec) with a jittered inter-stimulus interval of 55–66 sec. While in the photophobic state, bright light produced sharp pain localized to the left eye (pain intensity = 3/10), along with involuntary blinking. In the recovered state, the same paradigm produced the same frequency of blinking, but without pain.

Imaging was conducted using a 3T Siemens Trio scanner (Erlangen, Germany) with a phased array head coil. For anatomical scans, a sagittal three-dimensional T1-weighted scan (MPRAGE) was performed. For functional scans, a Gradient Echo (GE) echo planar imaging (EPI) sequence (TE/TR = 30/3000 ms; flip angle  = 90°; field of view  = 22.4 cm; slice thickness  =  contiguous 3.5 mm; in-plane resolution  = 3.5 mm) was performed, with 202 volumes captured for each scan.

**Figure 1:**
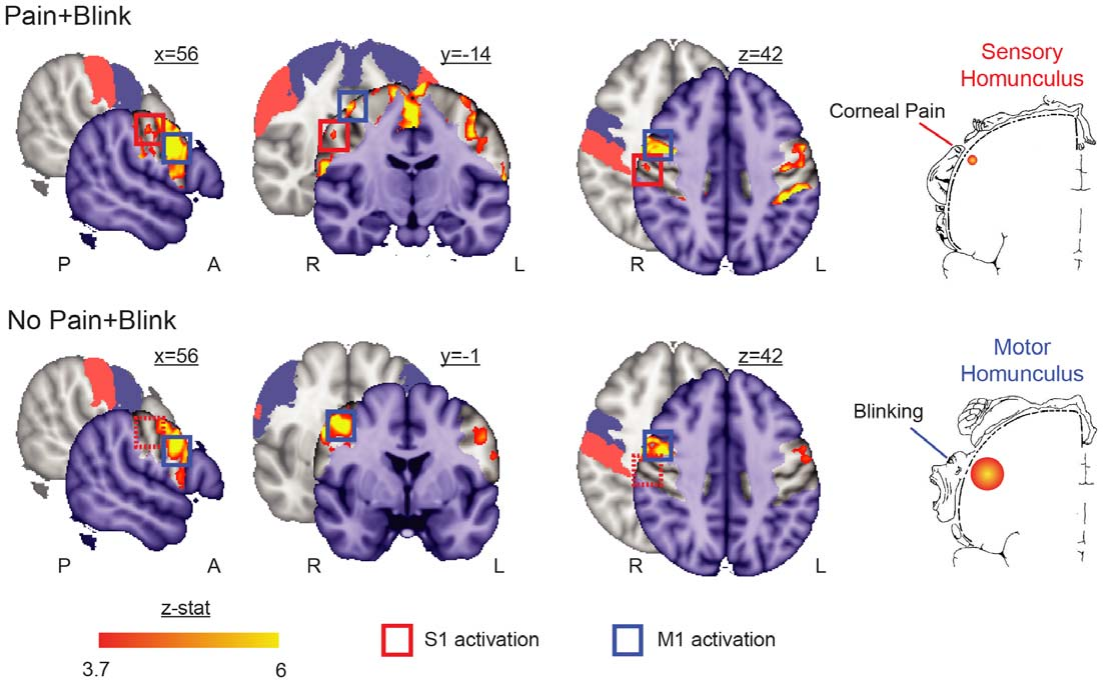
Somatotopic activation triggered by combined corneal pain and eye blink. The Pain+Blink condition activated contralateral S1 (Max zstat  = 4.9 at 56, −14, 43) and bilateral M1 (Max zstat  = 9.9 at 52, 2, 31) in regions corresponding to the eye in the Penfield sensory and motor homunculi [19] (p<0.0001, uncorrected for multiple comparisons). The No Pain+Blink condition activated bilateral M1 (Max zstat  = 7.4 at 44, −4, 43), but not S1. Investigations were restricted to the non-shaded areas in the activation maps, which correspond to bilateral pre- (blue) and post-central gyri (red) as highlighted in the underlying brain slices and colored squares (dashed squares denote absent activation). Note that the boundaries of these probabilistic-defined areas overlap with other regions, such as supplementary motor area, middle frontal gyrus, and supramarginal gyrus. Of note, the supplementary motor area has previously been associated with voluntary blinking [13], and was active in both conditions along the midline of coronal slice y = −14 (only shown for Pain+Blink in figure). A = anterior; L = left; P = posterior; R = right.

Functional imaging datasets were processed and analyzed using FEAT (FMRI Expert Analysis Tool) Version 5.98, part of FSL 4.1.1 (FMRIB's Software Library, www.fmrib.ox.ac.uk/fsl). For details, refer to [5]. The functional scan was affine registered to the T1 scan with FLIRT (FMRIB's Linear Image Registration Tool), followed by nonlinear registration to MNI152 space with FNIRT (FMRIB's Nonlinear Image Registration Tool). Given that our aim was to localize the cortical representation of the cornea in the somatosensory homunculus, we used a region of interest (ROI) approach to anatomically restrict the analysis to S1 and M1. These ROIs were identified based on the Harvard-Oxford Cortical Structural Atlas boundaries for the respective postcentral and precentral gyri (>25% probabilistic likelihood). This ROI approach differs from the whole-brain approach employed by the previous case report [5]. Activation maps related to the presentation of the white slide were generated and thresholded to p<10^−4^ (uncorrected for multiple comparisons).

## Results

Bright light evoked different task conditions in the first (Pain+Blink) and second (No Pain+Blink) scans. During Pain+Blink, light produced significant activation in the middle of the superior-inferior extent of contralateral S1 (Figure 1), which corresponds to the rostral face as previously reported for fMRI brain somatotopic activations by innocuous brushing of different surfaces of the face [6]. Localization with regards to trigeminal distribution is not provided, as this representation model is highly variable across studies (see [6] for review). In both scans, blinking produced significant activation bilaterally in M1, with robust activations parallel to those observed in the primary somatosensory cortex with pain (Figure 1). Further details regarding pain-specific activation of brain structures during photophobia in this subject, such as trigeminal nucleus caudalis and anterior cingulate cortex, have been reported previously [5]. Note that the previous paper did not distinguish S1 activation from the “precentral gyrus” cluster, as the focal S1 activation was contiguous with the robust M1 activation (Figure 1).

## Discussion

By evaluating activation with and without corneal pain, we show that activation is present in contralateral S1 during pain. Given that the cornea is a small structure, we correlated its location with neighboring blink-related activation in the motor cortex. The latter activation was directly opposite to the somatosensory activation. Based on these data, we propose that the location of the corneal activation is in the S1 homunculus consistent with the location of the eye representation. This is also consistent with our prior studies on the somatotopic location of the face in human S1 cortex in response to innocuous brushing [6]. The cornea is a highly sensitive structure [7], [8], densely innervated by sensory fibers that transduce noxious stimuli [9]. We suggest that it is through these nociceptive fibers that the corneal representation is conveyed, as we have previously detected activation along the trigeminal nociceptive pathway in this dataset [5]. As is the case with other regions of the body, nerve damage to the cornea can result in chronic neuropathic pain conditions [10].

Eyelid closure depends on motor neurons that innervate the orbicularis oculi (OO) muscle. Using rabies virus, the projection of these motor neurons to M1 cortex has been described in non-human primates [11]. Neuroimaging findings suggest that precentral gyrus activates with voluntary blinking and during blink inhibition [12], [13], [14]. Across the central sulcus, eye muscle proprioception is represented in S1 [15], with bilateral activations extending into the motor and premotor cytoarchitectonic areas. Tonic proprioceptive inputs into the M1 have also been proposed [16]. However, during scanning, the individual's eyes were focused on a fixation cross and were not moving. Thus, S1 activations are unlikely to be attributed to proprioception. In addition, significant activation differences in pain and non-pain conditions further support a minimal contribution of proprioception.

In the Penfield sensory homunculus, the eye is located in the contralateral mid-cortical region, at the rostral region of the face representation [17]. We are unaware of any data (electrophysiological, magnetic encephalography, fMRI) that show resultant S1 neural activity during painful stimulation of the cornea. Our data appear to replicate the representation of the eye in the Penfield sensory homunculus, and are further supported by previous imaging studies evaluating the cortical representation of the face in human S1 [6], [18].

## Conclusions

We believe this is the first study to localize the cortical representation of painful stimulation of the cornea in S1. Note that our results may be specific to this particular sensitized state (photophobia), and may not apply to non-sensitized corneal afferents. These findings suggest that the cornea is an optimal stimulation site to evaluate peripheral and central mechanisms involved in the development of chronic pain. With recent advances in our understanding of the corneal pain system in the context of chronic pain [10], neuroimaging can tap into this corneal stimulation model to help unravel the relationship between S1 and brain mechanisms of pain.
